# Neutrophil Camouflaged Stealth Nanovehicle for Photothermal‐Induced Tumor Immunotherapy by Triggering Pyroptosis

**DOI:** 10.1002/advs.202207456

**Published:** 2023-03-26

**Authors:** Xuya Yu, Guozheng Xing, Shupei Sheng, Limin Jin, Yan Zhang, Dunwan Zhu, Lin Mei, Xia Dong, Feng Lv

**Affiliations:** ^1^ Tianjin Key Laboratory of Biomedical Materials Key Laboratory of Biomaterials and Nanotechnology for Cancer Immunotherapy Institute of Biomedical Engineering Chinese Academy of Medical Sciences & Peking Union Medical College Tianjin 300192 P.R. China

**Keywords:** nanovehicle, neutrophil hitchhiking, precise delivery, pyroptosis, tumor immunotherapy

## Abstract

The regulation of tumor immunosuppressive microenvironments via precise drug delivery is a promising strategy for preventing tumor recurrence and metastasis. Inspired by the stealth strategy, a stealthy nanovehicle based on neutrophil camouflage is developed to achieve precise delivery and tumor immunotherapy by triggering pyroptosis. The nanovehicle comprises anti‐CD11b‐ and IR820‐conjugated bovine serum albumin nanoparticles loaded with decitabine. Camouflaged by neutrophils, the nanovehicles achieve efficient tumor delivery by neutrophil hitchhiking owing to the biotropism of neutrophils for tumors. The fluorescent signal molecule, IR820, on the nanovehicle acts as a navigation monitor to track the precise delivery of the nanovehicle. The released decitabine upregulates gasdermin E, and laser irradiation activates caspase‐3, thereby resulting in pyroptosis, which improves the system's adaptive immune response. In a triple‐negative breast cancer animal model, it regulates the immunosuppressive microenvironment for effective tumor immunotherapy and induces a long‐lasting and strong immune memory to prevent lung metastasis.

## Graphical abstract

Stealthy nanovehicle based on neutrophil camouflage is developed to achieve precise delivery and tumor immunotherapy by triggering pyroptosis. Stealth nanovehicles comprise drug storage, launch drivers, navigation trackers, and control‐trigger systems. It causes pyroptosis to improve the system's adaptive immune response and regulate the immunosuppressive microenvironment for effective tumor immunotherapy. It further plays a critical role in preventing lung metastasis.

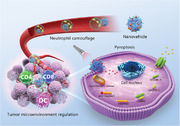

## 1 Introduction

The unique biological behavior, clinicopathological characteristics, and poor prognosis make triple‐negative breast cancer attract extensive attention from clinicians and researchers.^[^ ^1^ ^]^ Although surgery is the most important treatment for triple‐negative breast cancer, there is still a potential risk of tumor recurrence and metastasis after surgery. Tumor immunotherapy has made great progress for breast cancer, combating primary tumors and preventing tumor recurrence and metastasis.^[^ ^2^ ^]^ However, the lack of tumor antigens and the inability to effectively initiate adaptive immunity led to poor immunotherapy. Pyroptosis is a programmed cell death pathway that can effectively promote anti‐tumor immunity by releasing intracellular proinflammatory content and tumor antigen in the pyroptosis process.^[^ ^3^ ^]^ In particular, combining photothermal reagents and small‐molecule drugs promotes pyroptosis.^[^ ^4^ ^]^ Small‐molecule drugs can enhance the expression of gasdermin E (GSDME) in tumor cells. However, low‐power laser irradiation can activate caspase‐3 to cleave GSDME into the GSDME‐N domain and aggregate to form pores on cell membranes, resulting in pyroptosis. Nevertheless, it is important to consider co‐delivering them accurately to the tumor to reduce systemic toxicity and enhance therapeutic efficiency.

Nanomaterial delivery systems are important in co‐delivery and tumor‐targeted delivery.^[^ ^5^ ^]^ However, their delivery efficiency is still limited, and the safety of nanomaterials exposed to blood during systemic administration is also a risk factor.^[^ ^6^ ^]^ Living cell bionic delivery systems improve the precision of drug delivery efficiency and the safety of delivery vehicles owing to their biosafety and biotropism.^[^ ^7^ ^]^ The combination of living cells and nanomaterial delivery systems can achieve long circulation, high drug loading, and targeted delivery efficiency. In previous studies, we developed nano‐delivery systems loaded into living cell carriers, such as functional macrophages or platelets, for tumor‐targeted precision therapy.^[^ ^8^ ^]^ Among the living cell nano‐delivery systems, the in vivo cell hitchhiking‐based nano‐delivery system has a promising application prospect. Endogenous cells have natural delivery advantages such as good biocompatibility, low immunogenicity, and strong targeted self‐drive capability, avoiding the complex preparation and inactivation of living cell carriers in vitro.^[^ ^7^, ^9^ ^]^ In particular, the postoperative acute inflammatory microenvironment accelerates the aggregation of neutrophils,^[^ ^10^ ^]^ which facilitates the efficient loading and delivery of nanodrugs via neutrophils cell carriers.^[^ ^11^ ^]^ Moreover, studies reported that activated neutrophils could engulf more CD11b‐modified nanoparticles compared with other circulating blood cells such as monocytes, natural killer cells, granulocytes or macrophages.^[^ ^11b^ ^]^ Nano‐delivery systems based on neutrophil camouflage have greater potential for further treatment after breast cancer surgery.^[^ ^12^ ^]^ In this delivery process, the nano‐delivery system is cloaked by neutrophils in vivo. The biological tendency and protective ability of endogenous neutrophils can achieve targeted drug delivery and reduce the toxic side effects of nanomaterials.

**Figure 1:**
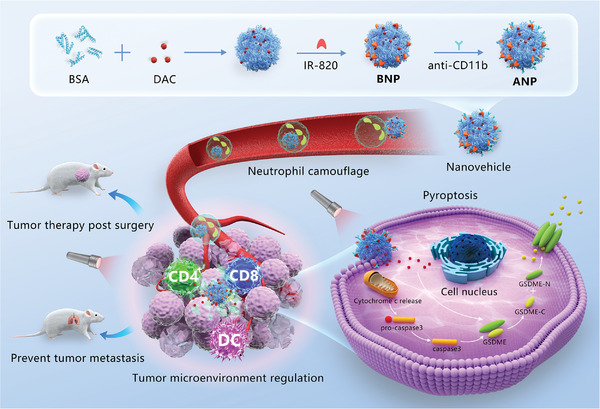
Scheme of neutrophil camouflaged stealth nanovehicle for photothermal‐induced tumor immunotherapy by triggering pyroptosis.

Inspired by the stealth strategy, we designed a stealthy nanovehicle based on neutrophil camouflage for precise delivery and tumor immunotherapy by triggering pyroptosis under fluorescent imaging navigation (**Figure** **1**). The nanovehicle comprised anti‐CD11b and IR820‐conjugated bovine serum albumin nanoparticles loaded with decitabine (DAC). By anchoring IR820 in nanoparticles, imaging navigation and photothermal‐enhanced pyroptosis for tumor immunotherapy could be achieved without additional fluorescent labeling.The delivery and treatment of the nanovehicle were divided into a series of processes, such as stealth, driven launch, exposure after reaching the destination, and precision attacks. After systematic administration, anti‐CD11b can target activated neutrophils in the blood to steal the nanovehicle. Stealth nanovehicles comprise drug storage, launch drivers, navigation trackers, and control‐trigger systems. Nanovehicles can achieve efficient tumor delivery by neutrophil hitchhiking owing to the biotropism of neutrophils to the postoperative inflammatory microenvironment.^[^ ^10a^ ^]^ The fluorescent signal molecule, IR820, on the nanovehicle acts as a navigation monitor to track the nanovehicle's precise delivery. In vivo imaging could monitor higher accumulation of nanoparticles at the tumor site duo to the rapid circulation of neutrophils in the blood. When the nanovehicle reaches the tumor area, the photothermal control system with IR820 allows it to escape from the cell carrier. Next, the released DAC upregulates GSDME and laser irradiation activates caspase‐3, which causes pyroptosis to improve the adaptive immune response of the system and regulate the immunosuppressive microenvironment for effective tumor immunotherapy, and further play a critical role in preventing lung metastasis.

## 2 Results and Discussion

**Figure 2:**
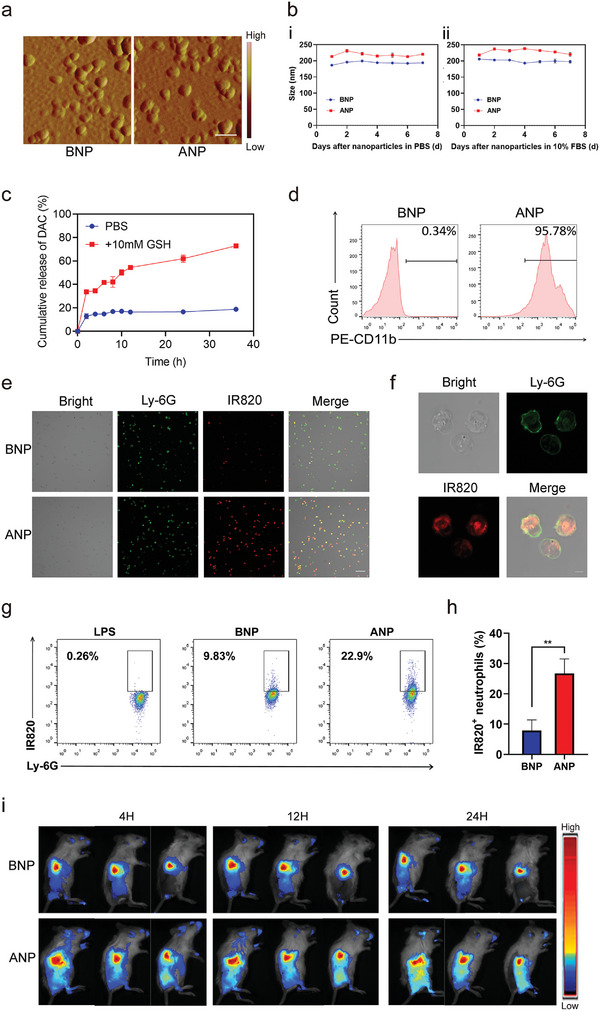
Nanoparticle characterization and role of antibody modification on neutrophil‐based tumor‐targeted drug delivery. a) AFM images. b) Stability studies: BNP and ANP in 7 d, *n* = 3. c) Comparative DAC release profile: in PBS and 10 mm GSH, *n* = 3. d) Anti‐CD11b coupling efficiency analysis by Flow Cytometer. e) Confocol images of neutrophils phagocytosing BNP and ANP, scale bar = 50 µm. Green: neutrophils stained with FITC‐anti‐Ly‐6G antibody; red: IR820 fluorescence signal from nanoparticles. f) Enlarged view of neutrophils phagocytosing ANP, scale bar = 5 µm. g) Representative flow cytometry analysis of nanoparticles targeting neutrophils in the blood of postoperative mice. h) Quantification of flow cytometric analysis of IR820^+^neutrophils ratio in the blood, Student's *t*‐test was performed, **p < 0.01, *n* = 3. i) In vivo imaging of postoperative mice 4, 12, and 24 h after BNP and ANP treatment.

First, we successfully prepared anti‐CD11b‐nanoparticles (ANP) nanovehicles in three steps: DAC loading, IR820 conjugation, and anti‐CD11b antibody modification of the albumin nanoparticles. Atomic force microscope (AFM images and particle dispersion index (PDI) results showed that both albumin nanoparticles without anti‐CD11b (BNP) and ANP had good dispersion (**Figure** **2**a; Figure S1, Supporting Information). Owing to the surface modification of anti‐CD11b antibodies, ANP has a larger particle size (216 nm) and stronger charge (_−_25.1 mV) than BNP nanoparticles (188 nm and _−_24.7 mV). There was no obvious change in the size of the nanoparticles within 7 days of storage, indicating good stability (Figure 2b). ANP exhibited fluorescence characteristics of IR820 (Figure S2, Supporting Information), which could ensure fluorescence imaging tracking capabilities.

Further photothermal imaging showed that ANP could rapidly heat up to 40 °C in 1 min and 45 °C in 5 min (Figure S3, Supporting Information). As the high content of reduced glutathione in the tumor microenvironment can break the disulfide bond of albumin in drug‐loaded nanoparticles, the in vitro drug release is promoted in the highly reduced glutathione release solution by 72% at 36 h (Figure 2c). The cell viability results show that the nanoparticle system has no significant killing effect on normal and tumor cells without activating the light control system, indicating the biosafety of the nanoparticle delivery system (Figure S4, Supporting Information).

Precise delivery of nanovehicles through neutrophil camouflage is the core of effective tumor therapy. Activation of neutrophils is a prerequisite for neutrophils to phagocytize CD11b‐modified nanoparticles in the blood. The postoperative acute inflammation not only activates neutrophils but also continuously recruits them to tumor during the total postoperative treatment phase of breast cancer, which offers the possibility of targeted delivery of neutrophil‐loaded cargo to postoperative tumor tissue due to the natural inflammatory tendency of neutrophils.^[^ ^10^ ^]^ The expression of CD11b molecules on the surface of activated neutrophils is remarkably increased; thus, anti‐CD11b antibody decoration would remarkably increase the recognition of nanoparticles by neutrophils.^[^ ^11b^ ^]^ Moreover, the molecular mechanism of CD11b‐modified nanoparticles hitchhiking neutrophil was addressed in aprevious report.^[^ ^11b^ ^]^In their opinion, it may be that neutrophils are activated earlier than other immune cells such as monocytes and macrophages in acute inflammation, leading to the specificity of neutrophils uptake of CD11b‐modified nanoparticles than other immune cells. In addition, the difference in the number of immune cells in the blood also causes the limited engulfing of CD11b‐modified nanoparticles by other immune cells such ase monocytes and macrophages. The neutrophil hitchhiking‐based tumor targeting delivery efficiency of CD11b‐modified nanoparticles will not be greatly affected by other immune cells. Here, we demonstrated the interaction between nanoparticles and neutrophils in vitro and in vivo and verified the effect of neutrophil‐mediated stealth nanovehicle‐targeted delivery. Flow cytometry data showed that 95% of the nanoparticles were successfully modified with CD11b antibody when the mass ratio of the antibody to nanoparticle was 1:80 (Figure 2d). Owing to the fluorescent properties of IR820, we observed a significant increase in the uptake of CD11b antibody‐modified nanoparticles by activated neutrophils relative to unmodified ones (Figure 2e).

Moreover, the internalization of ANP in activated neutrophils was observed at higher magnification (Figure 2f). For neutrophil‐mediated targeted delivery, nanoparticles must adhere to and be internalized by activated neutrophils in the blood after intravenous injection. To verify this process, we extracted neutrophils from the peripheral blood for analysis after injecting nanoparticles. The proportion of IR820‐positive neutrophils increased from 9.83% in the unmodified group (BNP) to 22.9% in the ANP group (Figure 2g). CD11b antibody modification greatly enhanced the affinity of the nanoparticles with activated neutrophils in the blood (Figure 2h), affirming the stealthy effect of nanoparticles based on neutrophil camouflage.

We then evaluated whether the acute inflammatory environment after tumor surgery promoted more stealth nanoparticles to be delivered to the tumor site with neutrophil hitchhiking by in vivo imaging. In vivo imaging results showed a higher accumulation of antibody‐modified nanoparticles at the tumor site than unmodified ones from 4 to 24 h because of the rapid circulation of neutrophils in the blood (Figure 2i). Ex vivo tissue imaging at 24 h displayed that the relative fluorescence intensity of postoperative tumor sites in the ANP group was 3.6 times higher than that in the BNP group (Figures S5 and S6, Supporting Information). Furthermore, stronger and more uniform fluorescence signals from IR820 of nanoparticles were also observed in frozen sections of tumor tissue in the ANP group (Figure S7, Supporting Information), suggesting that more neutrophil hitchhiking could facilitate nanoparticle infiltration into deeper tumor sites based on the biotropism of the neutrophils themselves. These results indicated that CD11b antibody modification caused more activated neutrophils in the blood to take up nanoparticles, thereby enhancing actively targeted drug delivery through neutrophil hitchhiking. In addition, in vivo thermal imaging demonstrated that the temperature of the tumor tissue increased to ≈45 °C within 5 min under low‐dose laser irradiation (Figure S8, Supporting Information). Therefore, efficient targeted delivery and photothermal effects guarantee photothermal‐induced tumor cell pyroptosis.

**Figure 3:**
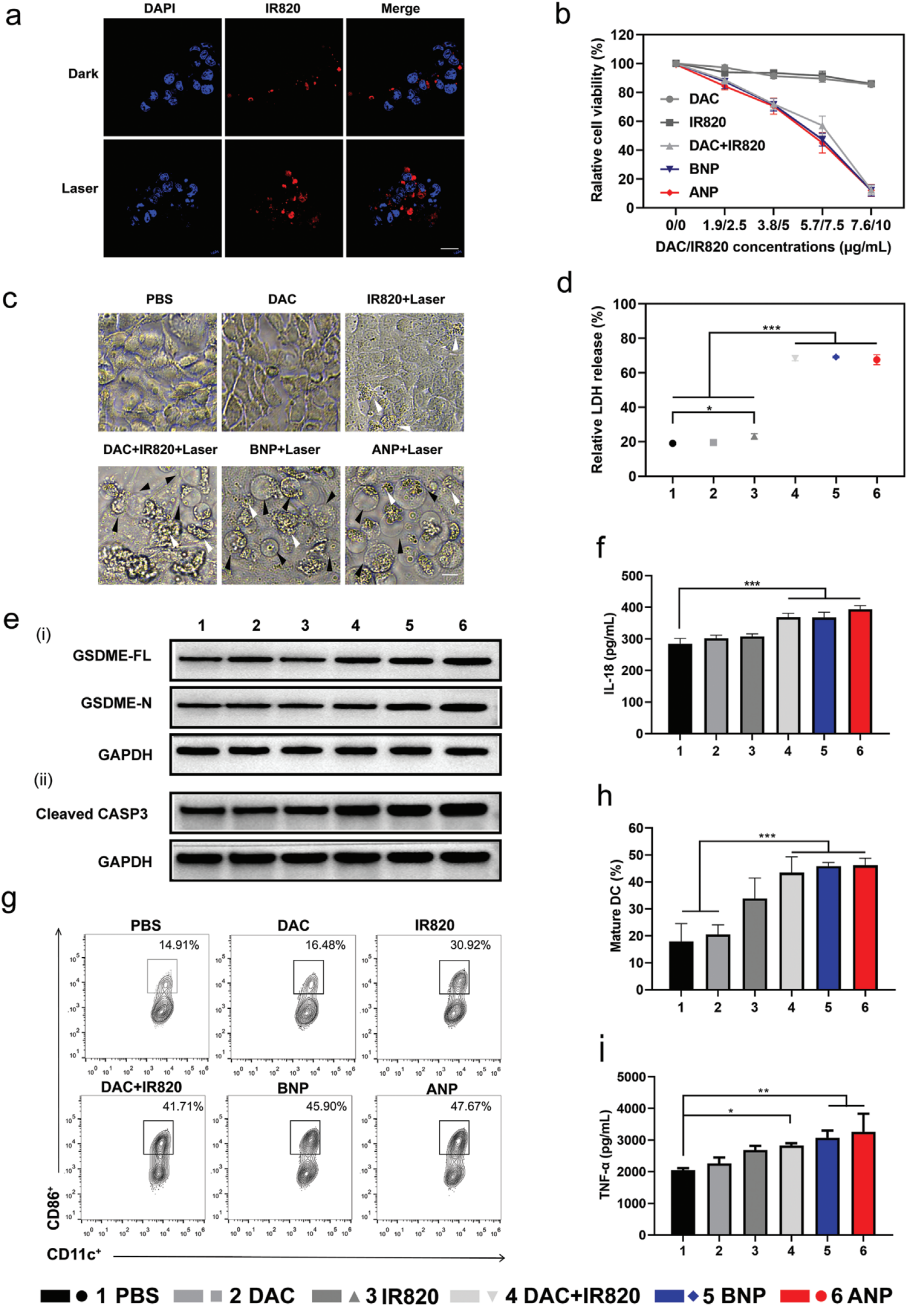
Photothermal‐induced tumor cells pyroptosis and its effect on DC maturation in vitro. a) Confocal images of photothermal‐controlled nanoparticles release from neutrophils into tumor cells, scale bar = 20 µm. Red: IR820 fluorescence signal from nanoparticles; Blue: nucleus stained with DAPI. b) Cell viability assessment of 4T1 cells after treating with PBS, DAC, IR820, IR820+DAC, BNP, and ANP in gradient concentrations under 808 nm laser irradiation, *n* = 3. c) Cell morphology was observed by inverted fluorescence microscopy under the concentration of DAC (3.8 µg mL^−1^) and IR820 (5 µg mL^−1^), scale bar = 10 µm. Black arrow: swollen pyroptosis cells; white arrow: apoptotic cells. d) Relative LDH release measurement. e) The expression of pyroptosis‐associated proteins e‐i) GSDME‐FL and GSDME‐N and e‐ii) Caspase‐3 were detected by western blot. f) The expression of IL‐18 in cell culture supernatants was measured simultaneously by ELISA. g) 4T1 cells with different treatments were co‐cultured with BMDCs to detect their effect on stimulating DC maturation. h) TNF‐*α* level in the BMDCs supernatant. All data were presented as mean ± SD. *P*‐values were calculated using one‐way ANOVA with Tukey correction, *p < 0.05, **p < 0.01, and ***p < 0.001, *n* = 3.

After the nanovehicle is delivered to the tumor site, exposure of neutrophils to the nanovehicle is the next important step in the therapeutic process. We investigated the process of photothermally controlled nanovehicle release from neutrophils into tumor cells using an in vitro cell co‐culture assay. The fluorescent signal in neutrophils was derived from IR820 on the nanocarrier. After laser irradiation, the fluorescence signal of IR820 appeared in the tumor cells, indicating that the nanoparticles migrated from the neutrophils to the tumor cells (**Figure** **3**a). In contrast, no significantly distributed fluorescence was observed in tumor cells without laser irradiation. These results suggested that a laser switch could promote the exposure of neutrophils to the nanovehicle and enhance the uptake of tumor cells.

The final stage of the nanovehicle was to attack tumor cells to exert the combined application of photothermal and demethylated drugs to promote pyroptosis for tumor immunotherapy. Pyroptosis, a programmed cell death mechanism, is closely associated with adaptive immunity.^[^ ^13^ ^]^ When cells undergo pyroptosis, the rapid release of cellular contents, including proinflammatory cytokines, stimulates a strong inflammatory response. Tumor antigens can activate antigen‐specific T cells, triggering a strong immunological effect.^[^ ^13^, ^14^ ^]^ However, the expression of GSDME, an important protein in pyroptosis, is usually disturbed by DFNA5 gene methylation, reducing the probability of pyroptosis.^[^ ^15^ ^]^ Therefore, we used DAC to demethylate DFNA5 to upregulate the expression of GSDME. Subsequently, IR820‐triggered photothermal stimulation activates the caspase‐3 pathway. GSDME is specifically cleaved to produce the GSDME‐N domain and aggregates to form pores on cell membranes, inducing cell pyroptosis. To verify our hypothesis, pyroptosis induced by photothermal stimulation combined with the DNA methyltransferase inhibitor DAC was confirmed by detecting cell morphology, the release of lactate dehydrogenase (LDH), proinflammatory cytokines, and protein expression.

Figure 3b shows the effect of photothermal‐induced pyroptosis on cell viability at different concentrations. Changes were observed in cell morphology after photothermal‐triggered pyroptosis using inverted microscopy. Parts of the 4T‐1 cell membranes in the BNP and ANP groups swelled, significantly different from photothermal‐induced apoptosis (Figure 3c). At the same time, photothermal effect could also promote apoptosis. Therefore, the combination of DAC and IR820 to kill tumor was the dual effect of pyroptosis and apoptosis, rather than the single effect of pyroptosis. However, free DAC treatment alone (DAC group) did not cause any changes in cell morphology. DAC treatment promoted GSDME expression without GSDME N‐terminal increase, which did not cause pyroptosis. DAC combined with laser irradiation (DAC+IR820) mainly induced apoptosis, possibly because of the instability of free DAC and its poor demethylation effect. Moreover, LDH release in DAC + IR820‐, BNP‐, and ANP‐treated cells significantly increased compared to the other groups (Figure 3d). Therefore, cell morphological changes and LDH release confirmed the occurrence of pyroptosis.

Western blotting was performed to analyze the expression of the pyroptosis‐related protein GSDME in 4T‐1 cells. DAC + IR820, BNP, and ANP treatment significantly increased GSDME‐FL expression. In contrast, the elevation in cleaved GSDME‐N expression was more remarkable in the BNP and ANP groups (Figure 3e‐i), which was conducive to pore‐forming activity. Correspondingly, cleaved caspase‐3 expression increased in all photothermal treatment groups (Figure 3e‐ii). In addition, the landmark proinflammatory molecule IL‐18 secretion significantly increased in the DAC + IR820, BNP, and ANP groups (Figure 3f). These data indicated that demethylated DAC upregulated GSDME expression, and photothermal‐activated caspase‐3 cleaved GSDME to GSDME‐N, leading to pyroptosis of tumor cells.

The leakage of cellular content and the release of cytokines and tumor antigens caused by pyroptosis activates dendritic cells (DCs) to present antigens to T cells for some immunological effects.^[^ ^13^, ^16^ ^]^ To illustrate this, bone marrow‐derived DCs (BMDCs) were used to evaluate their ability to stimulate DCs maturation after co‐incubation with 4T1 cells under different treatments in vitro. The results showed that DCs maturation induced by DAC + IR820, BNP, and ANP (CD11c^+^CD86^+^ DCs proportion was 41.7%, 45.9%, and 47.6%, respectively) was significantly higher than that induced by the IR820‐stimulated photothermal effect (30.9%) (Figure 3g,h). The secretion of TNF‐*α* in these three groups was also significantly enhanced (Figure 3i). This suggests that the dual role of pyroptosis and apoptosis from the combination of DAC and IR820 generates more inflammatory or immunostimulatory effects than photothermal‐induced apoptosis to trigger robust anti‐tumor immunological effects.

**Figure 4:**
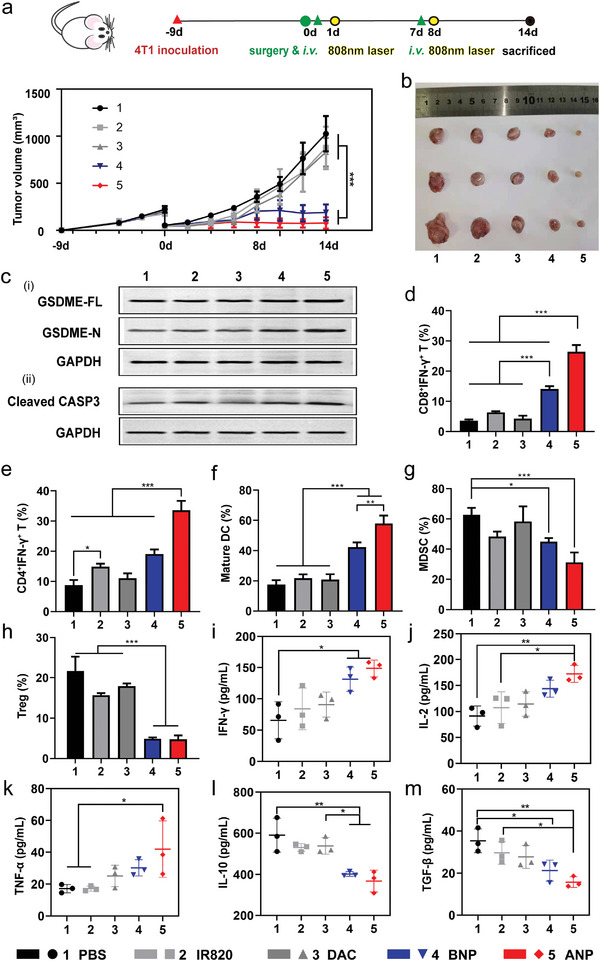
The effect of photothermal‐triggered pyroptosis on inhibiting tumor growth and regulating immunosuppressive microenvironment based on postoperative tumor model. a) Tumor growth curve after two cycles of drug injection, *p < 0.05, **p < 0.01, *n* = 5. b) Representative tumor pictures from different treatment groups 14 days after surgery, *n* = 3. c) Expression of pyroptosis‐associated proteins c‐i) GSDME‐FL and GSDME‐N and c‐ii) Caspase‐3 in tissue were measured by western blot. Flow cytometry assays determined the proportion of d) IFN‐*γ* ^+^CD8^+^ T cells, e) IFN‐*γ* ^+^CD4^+^ T cells, f) mature DC, g) MDSC, and h) Treg in tumor microenvironment. Cytokines contents including i) IFN‐*γ*, j) IL‐2, k) TNF‐ɑ, l) IL‐10, and m) TGF‐*β* in tumor were detected by ELISA. *p < 0.05, **p < 0.01, and ***p < 0.001, *n* = 3. All data were presented as mean ± SD. *P*‐values were calculated using one‐way ANOVA with Tukey correction.

The above studies have confirmed that CD11b antibody‐modified nanoparticles were adhered to and internalized by neutrophils in the blood and that more nanoparticles were targeted to the tumor by the natural inflammatory tendency of neutrophils. Next, we built a postoperative tumor model to study the effect of photothermal‐triggered pyroptosis on tumor growth inhibition and the regulation of the tumor immunosuppressive microenvironment. Female BALB/c mice (5–6 weeks) were obtained from Beijing Huafukang Biotechnology Co., Ltd (Beijing, China) and conducted in accordance with protocols approved by the Institutional Laboratory Animal Ethics Committee and the Institutional Animal Care and Use Committee (IACUC) of Peking Union Medical College (IRM‐DWLL‐2022055). In our anti‐tumor protocol, the treatment was postoperative administration plus laser irradiation (**Figure** **4**a). According to our previous studies on photothermal immunotherapy,^[^ ^17^ ^]^ two doses and laser irradiation 1 week apart are more effective. The interval between laser irradiation and drug delivery is determined by the effectiveness of targeted drug delivery. Compared with other nanodelivery systems, our delivery system based on neutrophil hitchhiking can achieve more efficient targeted drug delivery and reduce the toxic side effects of nanomaterials. The tumor growth curve showed that after two dosing cycles, free DAC treatment (DAC group) failed to inhibit tumor growth, and low‐intensity laser irradiation only had a moderate photothermal therapeutic effect (IR820 group). However, the tumor was effectively suppressed in the BNP and ANP groups, especially with the ANP treatment. We found an approximately 12‐fold decrease in tumor weight (Figure 4b; Figure S9, Supporting Information) in the ANP group relative to the PBS group on Day 14 after treatment. This demonstrates that the neutrophil hitchhiking delivery system effectively inhibited the growth of postoperative residual tumors and prevented their recurrence by initiating the tumor cell pyroptosis procedure. At the same time, body weight monitoring data (Figure S10, Supporting Information) and hematoxylin‐eosin (HE) staining (Figure S11, Supporting Information) of the major organs as well as complete blood count analysis (Table S1, Supporting Information) confirmed the biosecurity of this delivery system. No significant pathological changes or lesions occurred in the main organs for all therapeutic groups. Although this nanodelivery system relied on neutrophils hitchhiking in the blood, there were no obvious abnormalities in hematological indicators in the therapeutic groups.

Western blot analysis confirmed the pyroptosis process after treatment. Although the increase in GSDME‐FL expression in vivo was not as remarkable as in vitro in the BNP and ANP groups, the GSDME‐N terminal expression in both groups was significantly higher than in the other treatments (Figure 4c‐i). We also observed increased cleaved caspase‐3 expression (Figure 4c‐ii), indicating that photothermal stimulation effectively cleaved GSDME‐FL to the GSDME‐N terminus. These results suggest that pyroptosis occurs in tumor tissues and plays an important role in tumor suppression.

To evaluate the influence of photothermal‐triggered pyroptosis on the tumor microenvironment, we determined the proportion of various immune cells in the tumor tissue by flow cytometry. Among them, the proportion of IFN‐*γ*‐secreting effector T cells significantly increased in both BNP and ANP groups relative to the other treatment groups. Moreover, ANP‐triggered pyroptosis induced the highest percentage of effector T cells (CD8^+^IFN‐*γ* ^+^ T cells, 43.1%; CD4^+^IFN‐*γ* ^+^ T cells, 52.7%) through positive targeting and high tumor permeability (Figure 4d,e; Figure S12a,b, Supporting Information). Meanwhile, a higher maturity of intratumoral DC was observed in both the BNP and ANP groups (Figure 4f; Figure S12c, Supporting Information), allowing them to activate prime T cells more efficiently. In addition, the proportion of immunosuppressive cells, such as myeloid‐derived suppressor cells (MDSCs) (Figure 4g; Figure S13a, Supporting Information) and regulatory T cells (Tregs) (Figure 4h; Figure S13b, Supporting Information), decreased significantly in these two groups. It decreased moderately in the IR820‐induced photothermal effect group, and no significant change was observed in the DAC‐treated group. Similarly, the increased intratumoral cytokine IFN‐*γ* (Figure 4i), IL‐2 (Figure 4j), and TNF‐ɑ (Figure 4k) or decreased IL‐10 (Figure 4l) and TGF‐*β* (Figure 4m) levels also indicated an anti‐tumor microenvironment. These results indicate that photothermal stimulation alone could only moderately regulate the tumor microenvironment. In contrast, pyroptosis triggered by photothermal stimulation combined with DAC remarkably reversed the tumor‐promoting environment to a tumor‐suppressing environment through the release of various cell contents or cytokines, such that effector cells could better exert the anti‐tumor effect.

To confirm whether local pyroptosis can elicit a systemic immune response, IFN‐*γ*‐secreting killer T cells and their ability to kill tumor cells in vitro were detected. The proportions of IFN‐*γ* ^+^CD8^+^T cells and LDH release in the BNP and ANP groups were significantly higher than those in the other groups (Figure S14, Supporting Information), and a twofold increase in LDH level was observed in the ANP group relative to the BNP group (Figure S15, Supporting Information). Moreover, the proinflammatory cytokine levels (IL‐2, IL‐6, and TNF‐*α*) in the culture supernatant showed the same trend (Figure S16, Supporting Information). These results suggest that antibody modifications may be important in triggering stronger systemic immunity.

**Figure 5:**
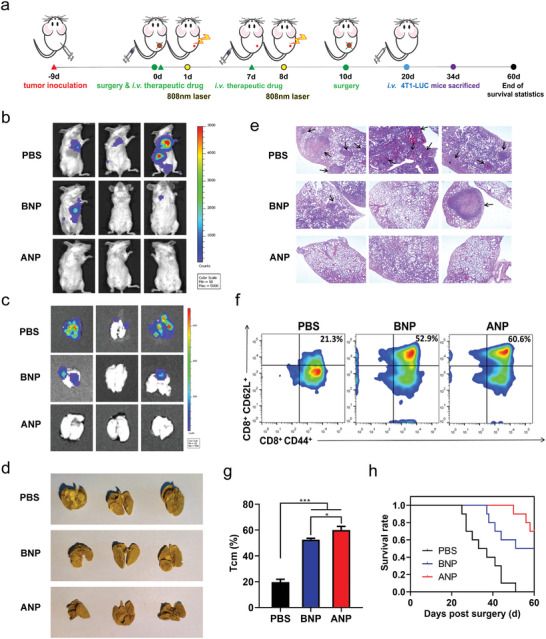
Photothermal‐triggered pyroptosis induced a long‐lasting immune memory to prevent tumor metastasis. a) Scheme of preventing lung metastases. b) Images of in vivo lung metastases. c) Isolated lung bioluminescence photograph. d) Small tumor nodules were clearly visible after fixation by Bouin's solution. e) H&E staining of fixed lungs, black arrow: tumor nodule. Scale bar = 500 µm. f,g) Splenocytes were stained with CD8, CD44, and CD62L to determine the effect of immunological memory, *p < 0.05, *n* = 3. Values on the bar graph were presented as mean ± SD. *P*‐values were calculated using one‐way ANOVA with Tukey correction. h) Survival rate curve of PBS, BNP, and ANP. Kaplan–Meier curve was used for survival curve analysis, *n* = 10.

In addition to effectively controlling in situ tumor recurrence after resection, we expect that the photothermal‐triggered pyroptosis can induce long‐lasting and strong immune memory to prevent tumor metastasis. 4T1‐Luc cells were injected (i.v.) into mice after the second dosing cycle to establish a tumor metastasis model (**Figure** **5**a). Two weeks after injection, a large bioluminescence signal appeared in the lungs of the PBS group, with only a minor metastasis in the BNP‐treated group, and no obvious signal was observed in the ANP group (Figure 5b). Moreover, the bioluminescence signal of the extracted lungs corresponded with the in vivo image results (Figure 5c). Bouin's solution fixation (Figure 5d) and HE staining (Figure 5e) results also confirmed distinct metastatic nodules in the lung; however, BNP‐induced pyroptosis significantly hindered pulmonary metastasis. In addition, an increase in memory T cells suggests that photothermal‐triggered pyroptosis prevents tumor metastasis by inducing long‐lasting and strong immune memory (Figure 5f,g). The ANP‐treated mice achieved the highest survival rate (Figure 5h).

Pyroptosis, a non‐apoptotic regulated cell death pathway, has multilevel relationships with systemic immune responses and is critical in tumor immunotherapy.^[^ ^18^ ^]^ New research has suggested that whether pyroptosis could suppress tumors largely depends on the tumor environment.^[^ ^19^ ^]^ Therefore, we applied a low‐dose photothermal effect to trigger pyroptosis. Our previous reports demonstrated that the photothermal effect could induce tumor immunogenic death to activate adaptive immunity and regulate the tumor microenvironment,^[^ ^20^ ^]^ and photothermal damage combined with photothermal‐triggered pyroptosis remarkably increases the motivation to activate systemic immunity. In addition, the danger signals released from photothermal‐triggered pyroptosis would recruit more anti‐tumor immune cells. At the same time, CD8^+^ T cells can also inhibit tumors by inducing ferroptosis and pyroptosis;^[^ ^21^ ^]^ thus, forming a positive feedback network of pyroptosis and anti‐tumor immunity.

## 3 Conclusion

In summary, we successfully prepared a stealthy nanovehicle based on neutrophil camouflage to achieve tumor immunotherapy by triggering pyroptosis. Owing to the biotropism of neutrophils to the postoperative inflammatory microenvironment, the nanovehicles achieved efficient tumor delivery by neutrophil hitchhiking. The released DAC upregulated GSDME, and laser irradiation activated caspase‐3, resulting in pyroptosis to improve the system's adaptive immune response. It regulated the immunosuppressive microenvironment for effective tumor immunotherapy and induced long‐lasting and strong immune memory to prevent lung metastasis. The interaction between pyroptosis mechanisms and anti‐tumor immunity was complex but our study only provided a new strategy for exploring nanomaterial‐mediated photothermal‐triggered pyroptosis for cancer immunotherapy.

## Conflict of Interest

The authors declare no conflict of interest.

## Supporting information

Supporting Information

Supporting Information

## Data Availability

The data that support the findings of this study are available in the Supporting Information of this article.
