# Supplementary material for: Neutrophil Camouflaged Stealth Nanovehicle for Photothermal‐Induced Tumor Immunotherapy by Triggering Pyroptosis
Source: Adv Sci (Weinh). 2023 Mar 26;10(15):2207456. doi: 10.1002/advs.202207456 (PMC10214253; doi:10.1002/advs.202207456)
Supplement: Supplementary file 1 — Supporting Information [file ADVS-10-2207456-s001.pdf]

ADVANCED SCIENCE
Open Access

Supporting Information

for *Adv. Sci.*, DOI 10.1002/advs.202207456

Neutrophil Camouflaged Stealth Nanovehicle for Photothermal-Induced Tumor Immunotherapy by Triggering Pyroptosis

Xuya Yu, Guozheng Xing, Shupei Sheng, Limin Jin, Yan Zhang, Dunwan Zhu, Lin Mei, Xia Dong* and Feng Lv**

Supporting information

Neutrophil Camouflaged Stealth Nanovehicle for Photothermal-Induced Tumor Immunotherapy by Triggering Pyroptosis

Xuya Yu, Guozheng Xing, Shupei Sheng, Limin Jin, Yan Zhang, Dunwan Zhu, Lin Mei,Xia Dong*, and Feng Lv**

X.Yu, G.Xing, S. Sheng, L. Jin, Dr. Y. Zhang, Prof. D. Zhu, Dr. X. Dong, Prof. L.Mei, Prof. F. Lv

Tianjin Key Laboratory of Biomedical Materials, Key Laboratory of Biomaterials and Nanotechnology for Cancer Immunotherapy, Institute of Biomedical Engineering, Chinese Academy of Medical Sciences & Peking Union Medical College, Tianjin, 300192, PR China

E-mail: meilin@bme.pumc.edu.cn (L. Mei)

E-mail: dongxia@bme.pumc.edu.cn (X. Dong)

E-mail: lvfeng@bme.pumc.edu.cn (F. Lv)

1. Materials

BSA was purchased from Biotopped (Beijing, China). New indocyanine green (IR820), decitabine (DAC), reduced glutathione (GSH) and sulfosuccinimidyl 4-(N-maleimidomethyl)cyclohexane-1-carboxylate (Sulfo-SMCC) were bought from Aladdin Industrial Corporation (Shanghai, China). Tris(2-carboxyethyl)-phosphine hydrochloride (TCEP) was obtained from Sigma-Aldrich. Anti-CD11b antibody (aCD11b) was obtained from Biolegend (San Diego, USA). GM-CSF and IL-4 were purchased from Peprotech (Rocky Hill, NJ, USA). MTS and LDH assay kit were purchased from Beyotime Biotechnology Co., Ltd (Shanghai, China). Mouse bone marrow neutrophil isolation solution kit was obtained from Solarbio Technology Co., Ltd (Beijing, China). All other antibodies and ELISA kits were purchased from Ebioscience (San Diego, USA).

2. Cell culture

4T1 and luciferase expressed 4T1 (4T1-LUC) murine mammary carcinoma cells were bought from Cell Resource Center, Shanghai Institute of Biotechnology, Chinese Academy of Sciences. 4T1 and 4T1-LUC cells were cultured in RPMI 1640 supplemented with 10% fetal bovine serum, 100 U/mL penicillin, and 100 μg/mL streptomycin in a humid atmosphere containing 5% CO_2 at 37 ℃. Bone-marrow-derived dendritic cells (BMDCs) were isolated from BALB/c mice according to previous reports[1].

3. Preparation and characterization

DAC loaded BSA NPs was prepared as described in ref[2]. Briefly, 40mg BSA

and 10mg GSH was added in 2mL deionized water, was adjusted to neutral. After 1h stirring, 100μL DAC solution was added and 2mL ethanol was dropped into the mixture for dialysis to obtain nanoparticles. IR820 decorated BSA NPs loaded with DAC (DAC@BSA-IR820 NPs, BNP) were prepared as follows. IR820 was activated as IR820-NHS for binding with BSA in a mass ratio of 1:25 at 37℃. Anti-CD11b antibodies were pre-treated with TCEP to expose the sulfhydryl group. BNP were first co-incubated with Sulfo-SMCC for 30 minutes and reacted with TCPE-prepared antibody for 1h at 4℃. The free antibody was removed by centrifugation to obtain anti-CD11b antibodies-linked DAC@BSA-IR820 NPs (DAC@BSA-IR820-CD11b NPs, ANP).

The morphology and dispersion of BNP and ANP was observed by a atomic force microscope (Multi-Mode 8, Veeco Instruments, USA). The size distribution and zeta potential of BNP and ANP was measured by a dynamic light scattering device (Malvern Instruments, Worcestershire, UK). The fluorescence spectra were detected by a fluorescent spectrophotometer (FluoroMax-4, HORIBA, Japan). Antibody coupling efficiency was determined using a flow cytometry (Calibur, BD, USA), using PE-aCD11b as a substitute. The loading and release ratio of DAC were analyzed by reverse-phase HPLC (Waters 1525, Waters, USA). The temperature changes of BNP and ANP was recorded by infrared thermometer (FLIR-E6390, FLIR, USA) under laser irradiation (808 nm, 1W/cm^2). The stability of BNP and ANP in PBS and 10% serum were evaluated by measuring particle size consecutively.

4. Photothermal induced cell pyroptosis in vitro

MTS assay was used to evaluate the cytotoxicity of BNP and ANP nanoparticles. To confirm the occurrence of cell pyroptosis, PBS, DAC, IR820, DAC+IR820, BNP and ANP (IR820: 0, 2.5, 5, 7.5, 10μg/mL; DAC: 0, 1.9, 3.8, 5.4, 7.6μg/mL) were incubated with 4T1 cells for 24h. Then, laser irradiation was applied in IR820, DAC+IR820, BNP and ANP groups (808 nm, 2 W/cm^2, 2min). Cell viability was detected to confirm the appropriate concentration for inducing cell pyroptosis by MTS assay after 24h. The morphology of pyroptosis 4T1 cells was taken by a inverted fluorescence microscope (DMi8 Manual, Leica, Germany) and the supernatants were collected to detect LDH release.

5. Tumor targeted delivery and photothermal effect

Neutrophils were isolated by mouse bone marrow neutrophil isolation kit and stimulated by LPS for 4h to prepare activated neutrophils. To verify the uptake of nanoparticles by activating neutrophils, BNP and ANP were co-incubated with activated neutrophils for 12h. Cells were collected by centrifugation and observed by laser scanning confocal microscope after staining with neutrophils specific PE-Ly6G antibody.

To verify whether nanoparticles can be released from neutrophils into tumor cells after irradiation, ANP-treated neutrophils were added to 4T1 cells and laser irradiation (808 nm, 2 W/cm^2, 5min), then stained nuclei with DAPI after PBS washing, the uptake of ANP by 4T1 cells was observed using laser scanning confocal microscope (LSM710, Carl ZEISS, Germany).

To further illustrate the role of antibody modification for neutrophils hitchhiking,

we induce an acute inflammatory tumor microenvironment by surgery, and PBS, BNP and ANP were intravenous (*i.v.)* injected 2h later. After that, neutrophils are isolated from the blood for analysis of nanoparticle uptake by flow cytometry.

To confirm the effect of tumor targeted delivery, PBS, BNP and ANP were *i.v.* injected 2h after tumor removing, *in vivo* imaging was used to observe the nanoparticle distribution utilizing a small animal live imaging system (CRi, USA). Temperature changes were recorded in tumor tissue within 5 minutes of laser irradiation after 24h i.v. injection of BNP and ANP. Mice were executed 24 hours after drug administration, the heart, liver, spleen, kidney and tumor were removed to further observe the nanoparticle distribution. Meanwhile, the scope of BNP and ANP infiltration in tumor was observed through frozen sections.

6. Photothermal induced pyroptosis for postoperative tumors therapy

1×10^6 4T1 cells were inoculated on the right side of 6-week-old female BALB/c mice. When the tumor volume was approximately $200mm^3$, the main tumor was surgically removed, leaving only about $50mm^3$ tumor blocks. Then the mice were randomly divided into five groups and *i.v.* administrated PBS, DAC, IR820, BNP and ANP (IR820: 0.5mg/kg; DAC: 0.19mg/kg) 24h after administration, the residual tumors were irradiated with NIR laser (808 nm, 0.5 W/cm^2). The injection and irradiation treatments were repeated twice with an interval of a week.Tumor volumes and body weight were measured every two days, tumor volumes were calculated as $L*W^2/2$ (L, longest dimension; W, shortest dimension). 14 days after surgery, the mice were sacrificed and tumors cells were extracted and stained for immune cells

analysis in the tumour microenvironment. The main organs were obtained for H&E staining. The leukogram and hematology in the whole blood was performed using an automated blood counter (BC-2800vet, Mindray, China) .

7. Flow cytometric staining and ELISA assay

For flow cytometric analysis, the cells were stained as follows: APC-anti-CD45, PE-anti-Gr-1 and PerCP-Cy5.5-anti-CD11c for MDSCs; APC-anti-CD45, FITC-anti-CD4 and PE-anti-FoxP3 for Tregs; FITC-anti-CD45, PE-anti-CD11c and APC-anti-CD86 for DCs; APC-anti-CD45, FITC-anti-CD4, PerCP-Cy5.5-anti-CD8a and PE-anti-IFN-γ for T cells in tumor; FITC-anti-CD8a, PerCP-Cy5.5-anti-CD4, PE-anti-CD62L and APC-anti-CD44 for memory T cells. In addition, cytokines level in cell culture supernatant and tumor tissue were detected according to the ELISA instructions.

8. Prevention of tumor lung metastasis

Establishing postoperative tumor models according to above method, the mice were randomly divided into three groups and treated with PBS, BNP and ANP. All tumors were removed after two cycles of treatment. 20 days after last administration, 1×10^5 4T1-Luc cells were *i.v.* injected to construct lung metastasis model. Two weeks later, lung metastases were observed in mice using a small animal live bioluminescent system (CaliperLifeSciences, IVIS, USA). Then, lung tissues were fixed by Bouin's solution to observe metastatic nodules, lung slices were stained with H&E for further examination. Mouse survival was monitored and recorded for 60 days.

9. Statistical analysis

All data was shown in the form of mean±standard deviation (S.D.). Group comparisons were performed using student t-test (group count=2) and one-way ANOVA followed by Dunnett's multiple comparisons test with Tukey correction (group count≥3). The significance were defined as *p<0.05, **p<0.01, ***p<0.001. Statistical analysis was performed with GraphPad Prism version 8.3.0 for Windows (GraphPad Software, San Diego, California USA).

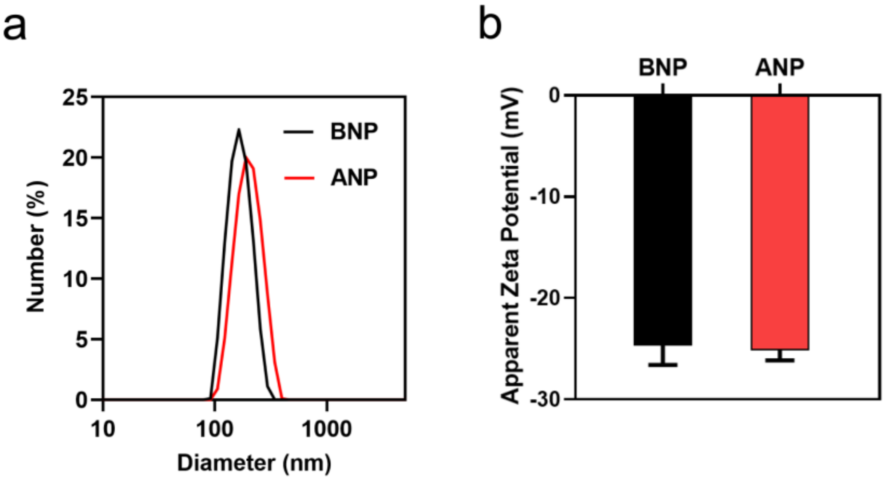

Figure S1. Size and zeta potential of BNP and ANP, n=3.

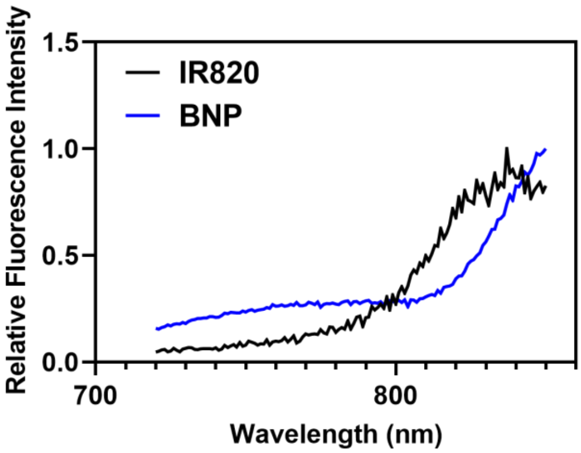

Figure S2. Fluorescence emission spectra of IR820 and BNP.

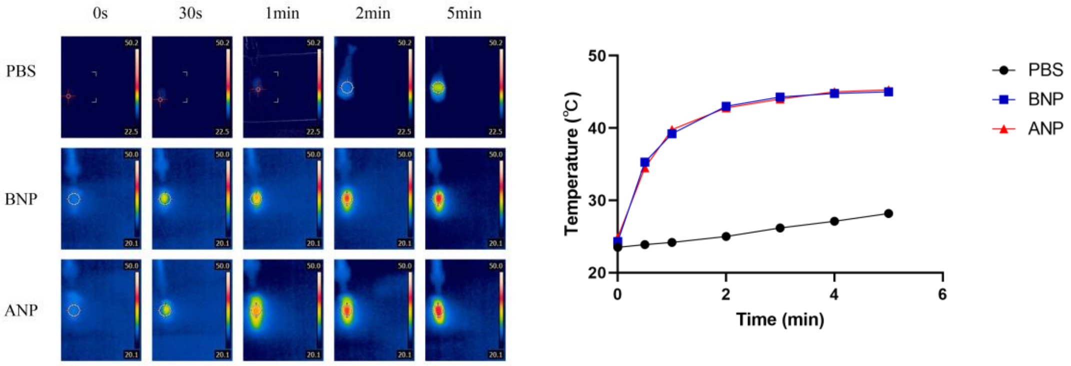

Figure S3. Heating curves of BNP and ANP upon laser irradiation as a function of irradiation time in 5min.

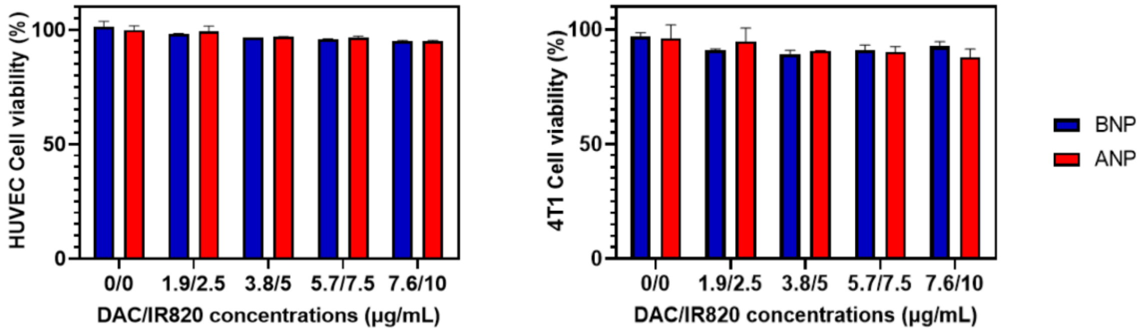

Figure S4. Dark toxicity of HUVEC and 4T1 after 24h co-incubation with BNP and ANP, n=3.

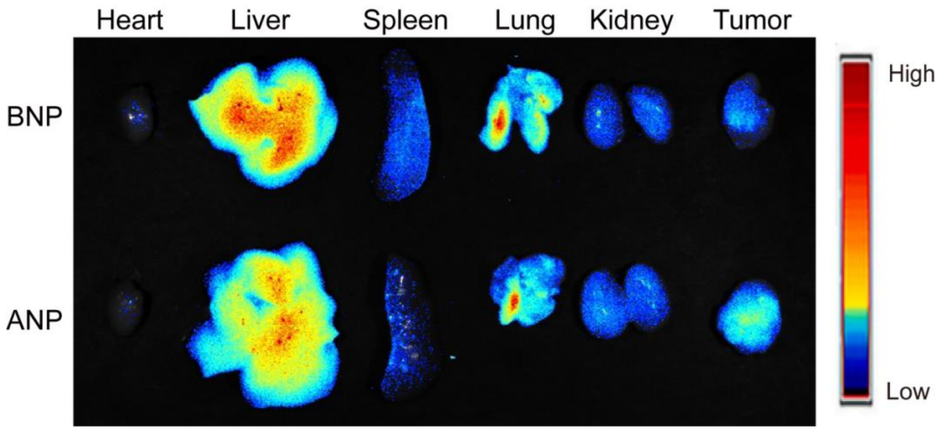

Figure S5. Ex vivo imaging of major organs and tumors 24h after BNP and ANP treatment.

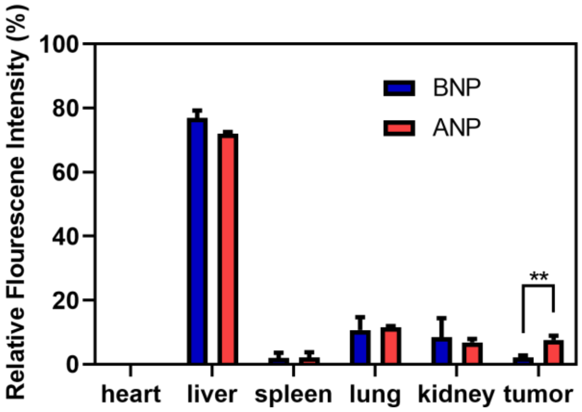

Figure S6. The relative flourescene intensity in major organs and tumors 24 hours after intravenous injection. Student t-test was performed, $^{**}p < 0.01$, n=3.

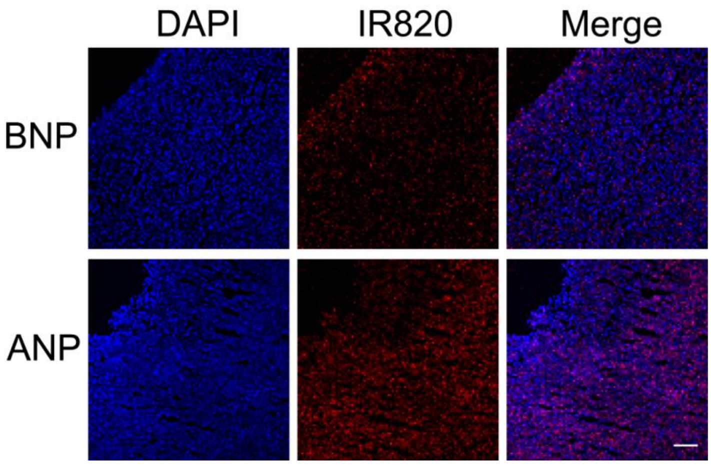

Figure S7. Corresponding confocal imaging of frozen tumor sections, scale bar = 100 μm. Red signal: nanoparticles; blue signal: nucleus stained with DAPI.

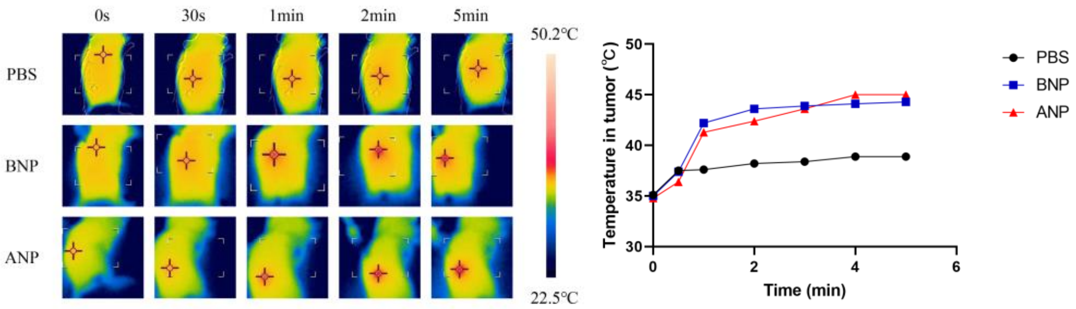

Figure S8. Heating curves of tumor site upon laser irradiation in 5min.

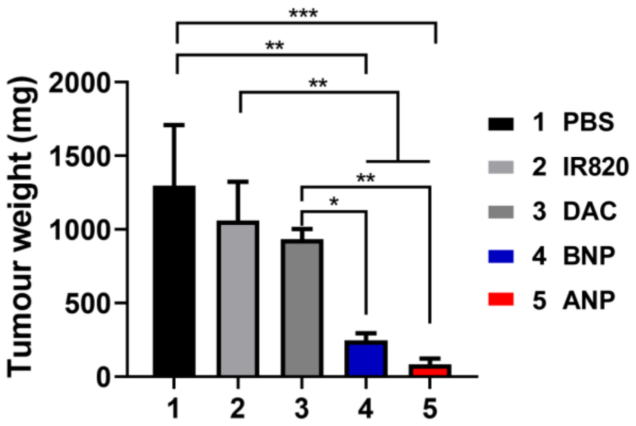

Figure S9. Tumour weight statistics for different treatment groups at 14 days after surgery, one-way ANOVA with Tukey correction was used,**p* < 0.05, ***p* < 0.01, ****p* < 0.001, n=3.

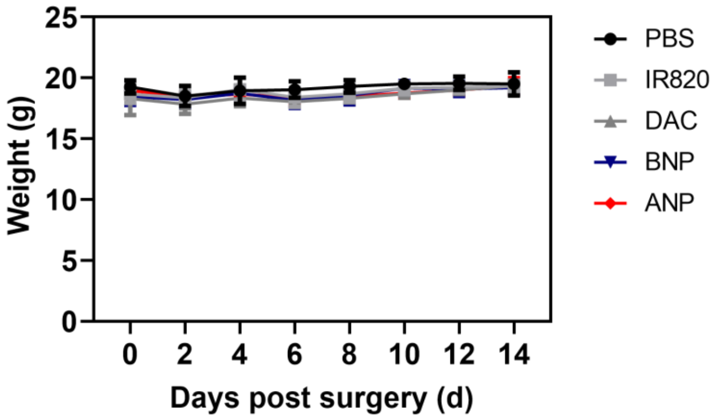

Figure S10. Body weight curve over time after surgery of removing tumors, n=5.

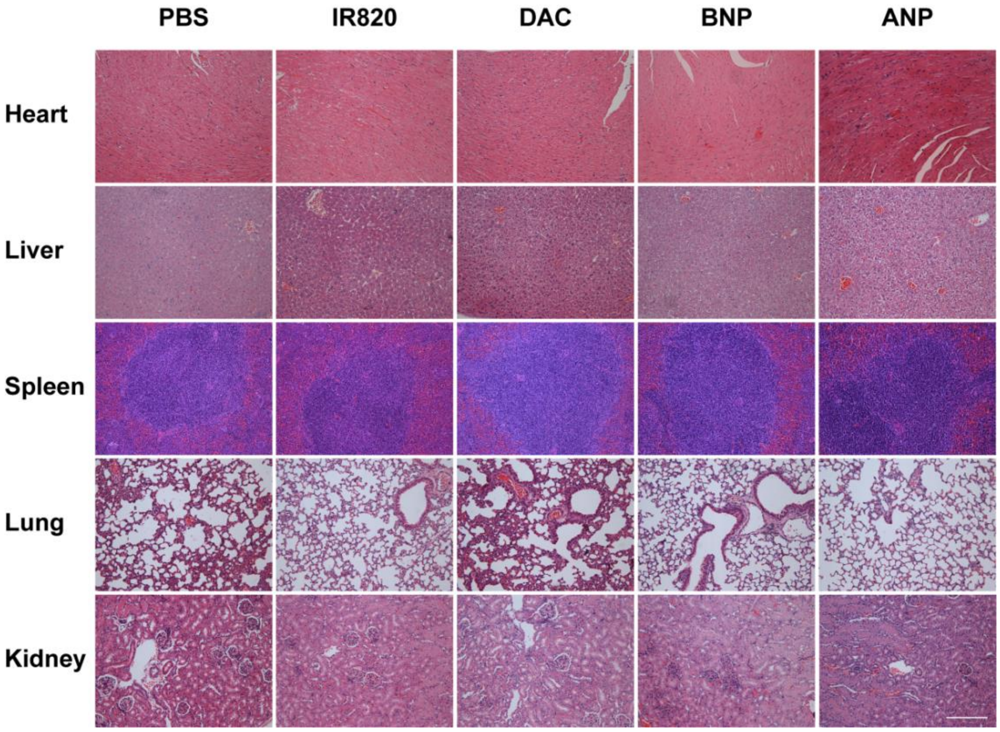

Figure S11. H&E staining of major organs, scale bar = 200μm.

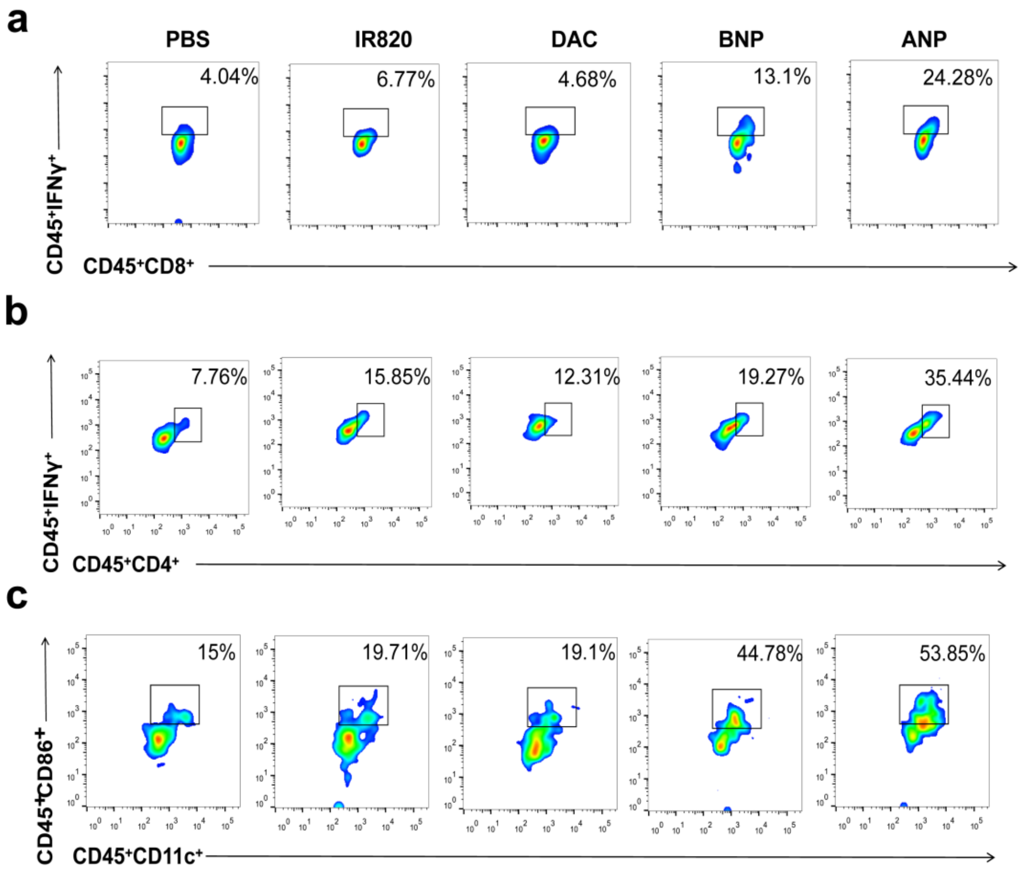

Figure S12. Flow cytometry analysis of representative immune cells that inhibit tumor growth in tumor microenvironment, (a) $CD8^{+}IFN\text{-}\gamma^{+}$ T cells; (b) $CD4^{+}IFN\text{-}\gamma^{+}$ T cells; (c) mature DCs.

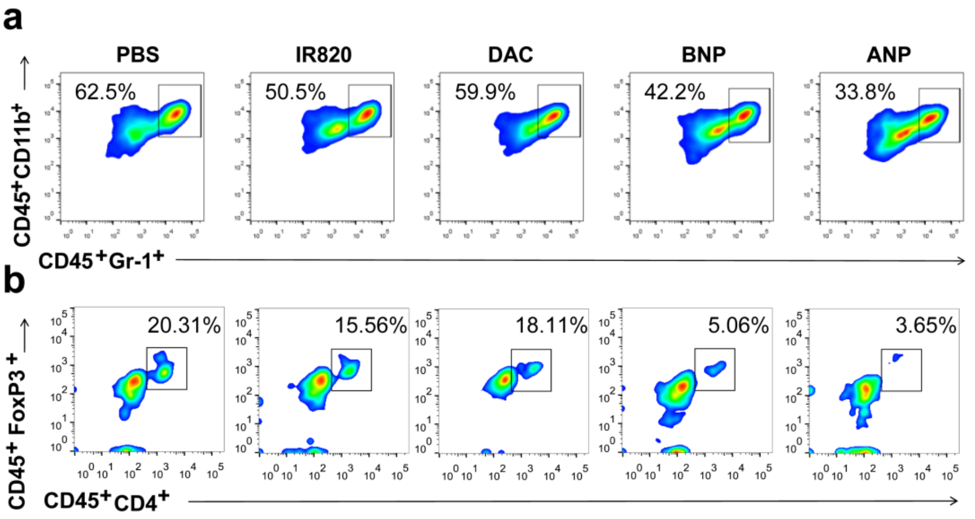

Figure S13. Flow cytometry analysis of representative immune cells that promote tumor growth in tumor microenvironment, (a) MDSCs; (b) Tregs.

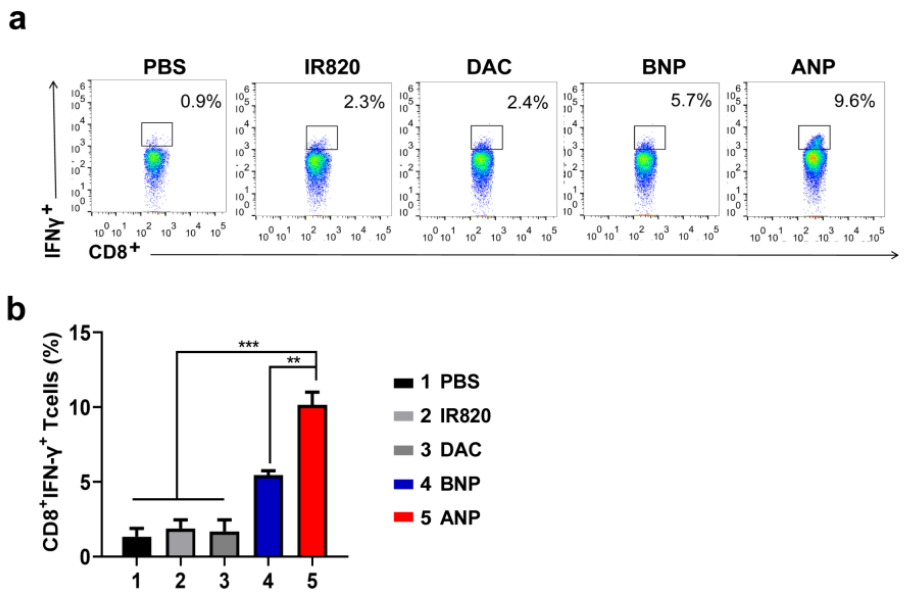

Figure S14. (a) Flow cytometry images of CD8$^+$IFN-γ$^+$ T cells after re-stimulating splenocytes with tumor antigen at 14d after surgery. (b) Corresponding statistical graph, one-way ANOVA with Tukey correction was performed, **p < 0.01, ***p < 0.001, n=3.

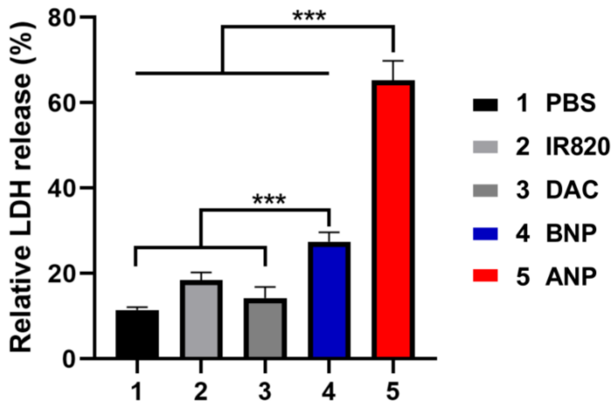

Figure S15. Supernatant relative LDH release after co-culture of splenocytes with 4T1 cells at a 20:1 ratio for 12 hours, one-way ANOVA with Tukey correction was applied, ***p < 0.001, n=3.

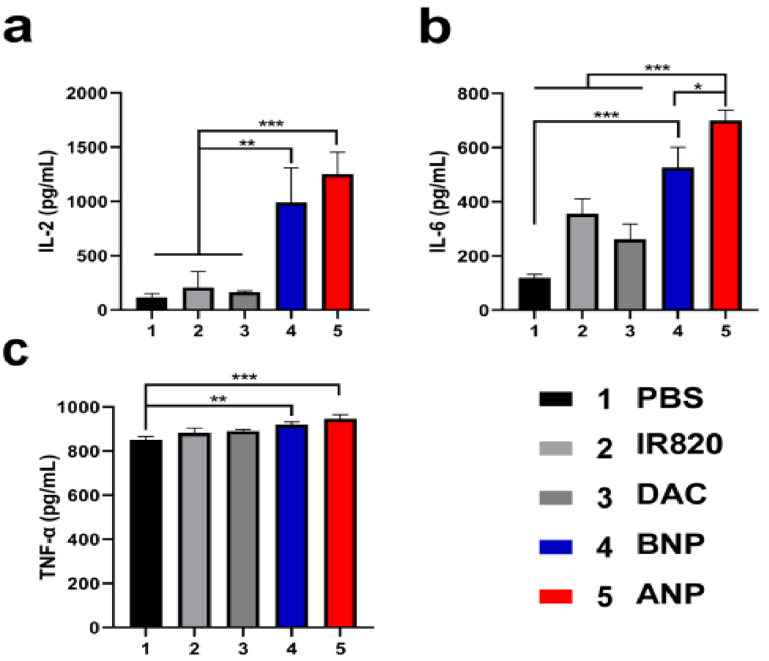

Figure S16. Splenocyte supernatants were collected and measured of cytokine levels by ELISA. (a) IL-2; (b) IL-6; (c) TNF-α. All data was presented as mean±SD. *P*-values were calculated using one-way ANOVA with Tukey correction, $*p < 0.05$, $**p < 0.01$, $***p < 0.001$, n=3.

Table S1 Complete Blood Count Report.

HEMATOLOGY	PBS	IR820	DAC	BNP	ANP	REF.RANGE
WBC	5.6±0.7	5.9±1.7	4.5±1.2	4.7±1	4.8±1.2	0.8-6.8
Lymph	4.7±0.8	4.5±1.4	3.5±1	3.6±0.8	3.8±0.9	0.7-5.7
Mon	0.2±0.1	0.1±0.1	0.1±0	0±0.1	0.1±0	0.0-0.3
Gran	1.3±0.4	1.3±0.2	0.9±0.2	1.1±0.2	0.9±0.3	0.1-1.8
Lymph%	80.5±1.1	76.1±2.8	76.9±3.9	75.9±1.1	79±1.3	55.8-90.6
Mon%	2±0.1	2±0.2	2.1±0.5	2±0.4	2.8±0.1	1.8-6.0
Gran%	17.4±1.2	22±2.7	21±3.4	22.1±1.4	18.2±1.3	8.6-38.9
RBC	9.3±0.2	9±0.9	9.3±0.1	9.3±0.1	9.2±0.4	6.36-9.42
HGB	141±5.6	142.7±0.6	141±4.6	132.7±9.7	141.3±5	110-143
HCT	44.1±0.2	43.6±1.9	44.3±0.3	43.8±1.3	43±1.4	34.6-44.6
MCV	48.5±0.2	48.2±0.7	48.2±0.2	48.3±1.2	48.5±1.4	48.2-58.3
MCH	16.4±0.5	16.2±0.6	16.5±0.3	16.6±0.3	16.3±0.3	15.8-19
MCHC	344.7±6	346.7±4.2	344.7±7.6	346.3±3.5	338±4	302-353
RDW	14.1±0.9	14.1±1.3	14.1±0.4	13.4±0.3	14.6±1.6	13.0-17.0
PLT	761±136.4	762.3±202.5	828.3±222.8	888.3±68.2	806±187.4	450-1590
MPV	4.7±0.3	5±0.3	4.8±0.3	5.3±0.5	4.8±0.1	3.8-6.0

References:

[1] X. Dong, J. Liang, A. Yang, Z. Qian, D. Kong, F. Lv, *Biomaterials* **2019**, 209, 111.

[2] W. Wang, Y. Huang, S. Zhao, T. Shao, Y. Cheng, *Chemical communications* **2013**, 49, 2234.
