# Supplementary material for: Neutrophil Camouflaged Stealth Nanovehicle for Photothermal‐Induced Tumor Immunotherapy by Triggering Pyroptosis
Source: Adv Sci (Weinh). 2023 Mar 26;10(15):2207456. doi: 10.1002/advs.202207456 (PMC10214253; doi:10.1002/advs.202207456)
Supplement: Supplementary file 2 — Supporting Information [file ADVS-10-2207456-s002.pdf]

ADVANCED SCIENCE
Open Access

Supporting Information

for *Adv. Sci.*, DOI 10.1002/advs.202207456

Neutrophil Camouflaged Stealth Nanovehicle for Photothermal-Induced Tumor Immunotherapy by Triggering Pyroptosis

Xuya Yu, Guozheng Xing, Shupei Sheng, Limin Jin, Yan Zhang, Dunwan Zhu, Lin Mei, Xia Dong* and Feng Lv**

The raw data for Western blots

1. The raw data for Western blots *IN VITRO* of Figure 3e

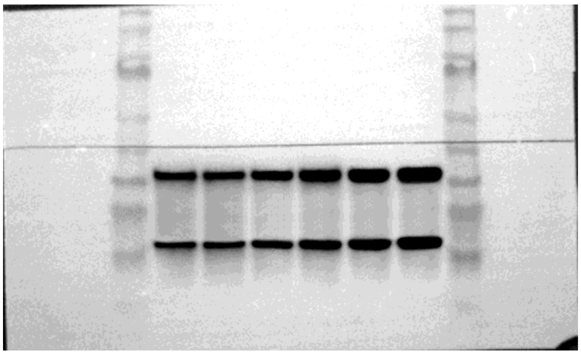

Up: Pro-caspase3
Below: Cleaved caspase3

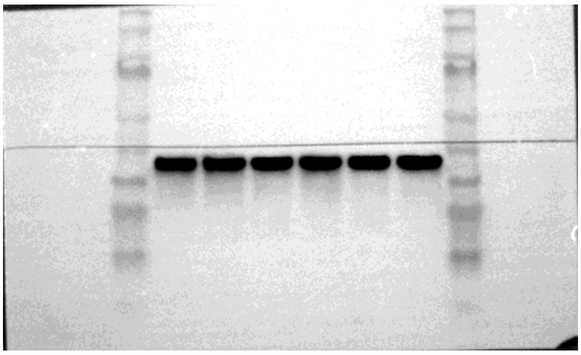

Caspase3-GAPDH

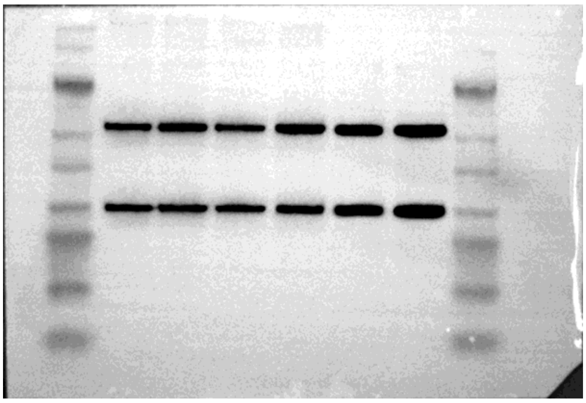

Up: GSDME-FL
Below: GSDME-N

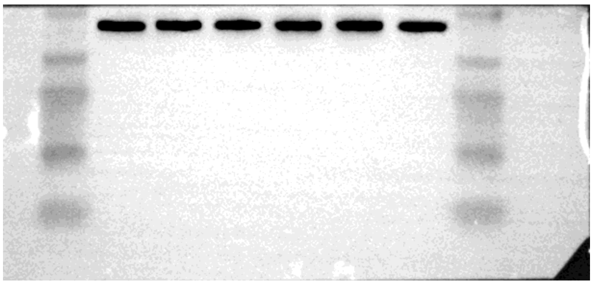

GSDME-GAPDH

2. The raw data for Western blots *IN VIVO* of Figure 4c

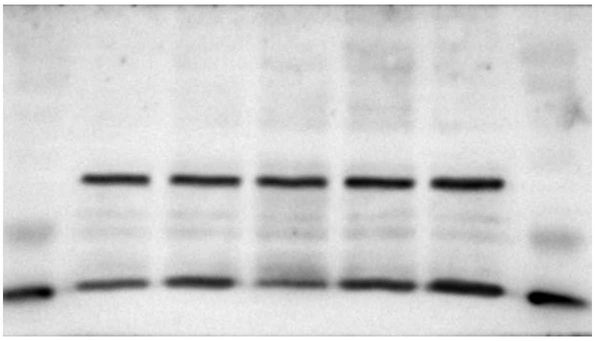

Up: Pro-caspase3
Below: Cleaved caspase3

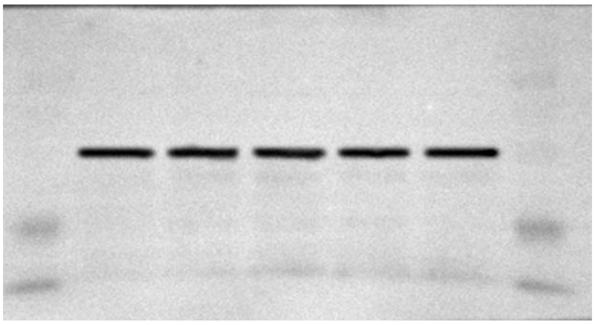

Caspase3-GAPDH

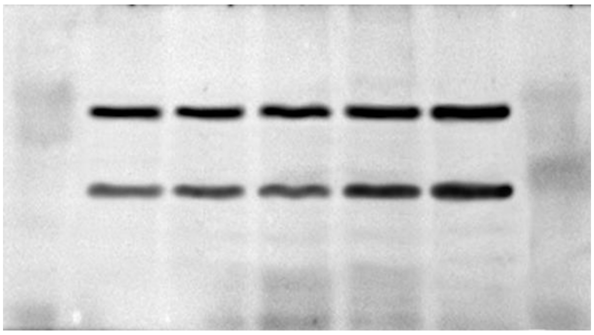

Up: GSDME-FL
Below: GSDME-N

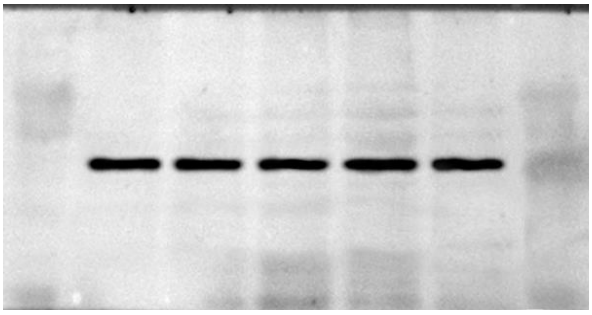

GSDME-GAPDH
